# The origin and evolution of air sacs in pterosaurs and their forerunners

**DOI:** 10.1111/joa.70030

**Published:** 2025-08-07

**Authors:** Tito Aureliano, Rodrigo T. Müller, Borja Holgado, Leonardo Kerber, Aline M. Ghilardi

**Affiliations:** ^1^ Department of Biological Chemistry, Programa de Pós‐Graduação Em Diversidade Biológica e Recursos Naturais Regional University of Cariri (URCA) Crato Brazil; ^2^ Diversity, Ichnology and Osteohistology Research Group (DINOlab) Federal University of Rio Grande Do Norte (UFRN) Natal Brazil; ^3^ AKCIT‐IMD Federal University of Rio Grande do Norte (UFRN) Natal Brazil; ^4^ Centro de Apoio à Pesquisa Paleontológica da Quarta Colônia Universidade Federal de Santa Maria (UFSM) São João Do Polêsine Brazil; ^5^ Museu de Paleontologia Plácido Cidade Nuvens Universidade Regional Do Cariri Santana do Cariri Crato Brazil; ^6^ Institut Català de Paleontologia Miquel Crusafont (ICP‐CERCA) Universitat Autònoma de Barcelona Cerdanyola del Vallès Catalonia Spain; ^7^ Museu Câmara Cascudo Universidade Federal do Rio Grande do Norte Natal‐RN Brazil

**Keywords:** anatomy, evolution, paleontology, respiratory system, zoology

## Abstract

Although the existence of postcranial pneumaticity and the inferred presence of air sacs connected to the lungs are well established in Pterosauria, the origin of this system in pterosaurs remains unclear. We investigated skeletal pneumaticity in the Triassic pterosauromorph *Venetoraptor* using microcomputed tomography, seeking insights into the origin of postcranial pneumaticity. Our analysis reveals distinct patterns of postcranial pneumaticity, providing insights into the evolution of the respiratory adaptations of pterosauromorpha. *Venetoraptor* exhibits a mosaic of pneumatic foramina and internal chambers in its vertebrae, suggesting the early evolution of an elaborate system of air sacs connected to the lungs, which suggests the presence of an elaborate respiratory system. These findings support the hypothesis that invasive air sacs predated true pterosaurs, providing advantages such as enhanced ventilatory efficiency, reduced skeletal mass, and increased mechanical strength, all crucial for powered flight. Our study underscores the significance of early pneumatic structures in shaping vertebrate flight evolution, positioning pterosauromorphs as one of the key intermediary lineages in the development of avian‐style respiratory systems.

## Graphical abstract

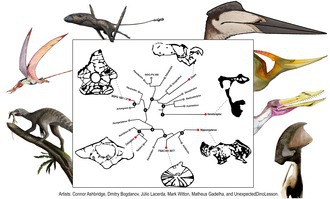

## 1 INTRODUCTION

Active flight evolved independently at least four times throughout Earth's history. Pterosaurs were the first flying vertebrates, appearing over 216 million years ago, during the Late Triassic (Butler et al., 2009; Jagielska & Brusatte, 2021). [Correction added on 18 September 2025 after first online publication: The number of times has been updated to “four” from “five” in this sentence.] Key adaptations to flight include high metabolic demand necessitating efficient lung ventilation and a low‐density body (Maina, 2005; Proctor & Lynch, 1993; Schachner et al., 2024). Air sacs and the diverticula that branch from them present in crown group birds permeate soft organs and penetrate the skeleton, enhancing the total volume of captured air through unidirectional airflow, and decreasing body density. This pneumatization not only reduces skeletal mass but can also increase the bone's mechanical strength (Lawson et al., 2025; Maina, 2006; O'Connor, 2004; Snively et al., 2006).

Fossil evidence of pneumatic diverticula is limited to traces they leave in the axial and appendicular bones, in the form of pneumatic fossae and foramina. Pneumatic foramina are typically continuous with large pneumatic spaces inside of bones; skeletal elements with such openings are described as having postcranial skeletal pneumaticity (PSP hereinafter) (Britt, 1993). Unambiguous PSP is determined through the detection of enlarged foramina or fossae connected with internal chambers in vertebrae, ribs, or appendicular elements (O'Connor, 2006). The development of invasive air sacs is thought to be opportunistic, following the preexisting vascular network throughout ontogeny, playing a crucial role in shaping vertebral internal architecture (Britt, 1993; Taylor & Wedel, 2021; Wedel et al., 2000).

Evidence of unambiguous PSP from the fossil record is restricted to select groups of ornithodirans: neotheropods (Aureliano et al., 2024; Benson et al., 2012; Gianechini & Zurriaguz, 2021; Smith et al., 2021; Wedel, 2009), plateosaurian sauropodomorphs (Aureliano et al., 2021; Aureliano et al., 2023; Cerda et al., 2012; Wedel, 2003; Wedel et al., 2000; Windholz et al., 2020; Zurriaguz, 2024), and pterosaurs (Buchmann et al., 2021; Butler et al., 2009; Smith et al., 2004; Williams et al., 2021). Evidence suggests at least three independent origins of PSP, one for each of these lineages (Aureliano et al., 2022). However, it remains unclear whether the invasive air sacs crucial for flight in pterosaurs were present in their pterosauromorph ancestors.

To address this question, we conducted high‐resolution microcomputed tomography (micro‐CT) analyses of the largerpetid pterosauromorph *Venetoraptor* (Müller et al., 2023), from the Upper Triassic of South Brazil, to evaluate the presence of unambiguous PSP. By comparing its microanatomy with several pterosaurs, we aimed to reconstruct the evolutionary trajectory of invasive air sacs within Pterosauromorpha.

## 2 MATERIALS AND METHODS

### 2.1 Institutional abbreviations

CAPPA/UFSM, Centro de Apoio à Pesquisa Paleontológica da Quarta Colônia, Universidade Federal de Santa Maria, São João do Polêsine, Rio Grande do Sul state, Brazil; BYU, Brigham Young University, Provo, United States of America; LPP‐UFRN, Paleontology and Paleoecology Laboratory, Universidade Federal do Rio Grande do Norte, Natal, Rio Grande do Norte state, Brazil; FSAC‐KK, Faculté des Sciences de Casablanca, Morocco; NHMUK, Natural History Museum, London, England; NMS, National Museums Scotland, Edinburgh, Scotland; SNSB/BSPG, Staatliche Naturwissenschaftliche Sammlungen Bayerns / Bayerische Staatssammlung für Paläontologie und Geologie, Munich, Germany; ULBRA, Centro de Apoio à Pesquisa Paleontológica da Quarta Colônia, Universidade Federal de Santa Maria, São João do Polêsine, Rio Grande do Sul state, Brazil (previously Museu de Ciências Naturais, Universidade Luterana do Brasil, Canoas, Brazil); ZPAL, Institute of Paleobiology of the Polish Academy of Sciences in Warsaw, Poland.

### 2.2 Specimens

**FIGURE 1:**
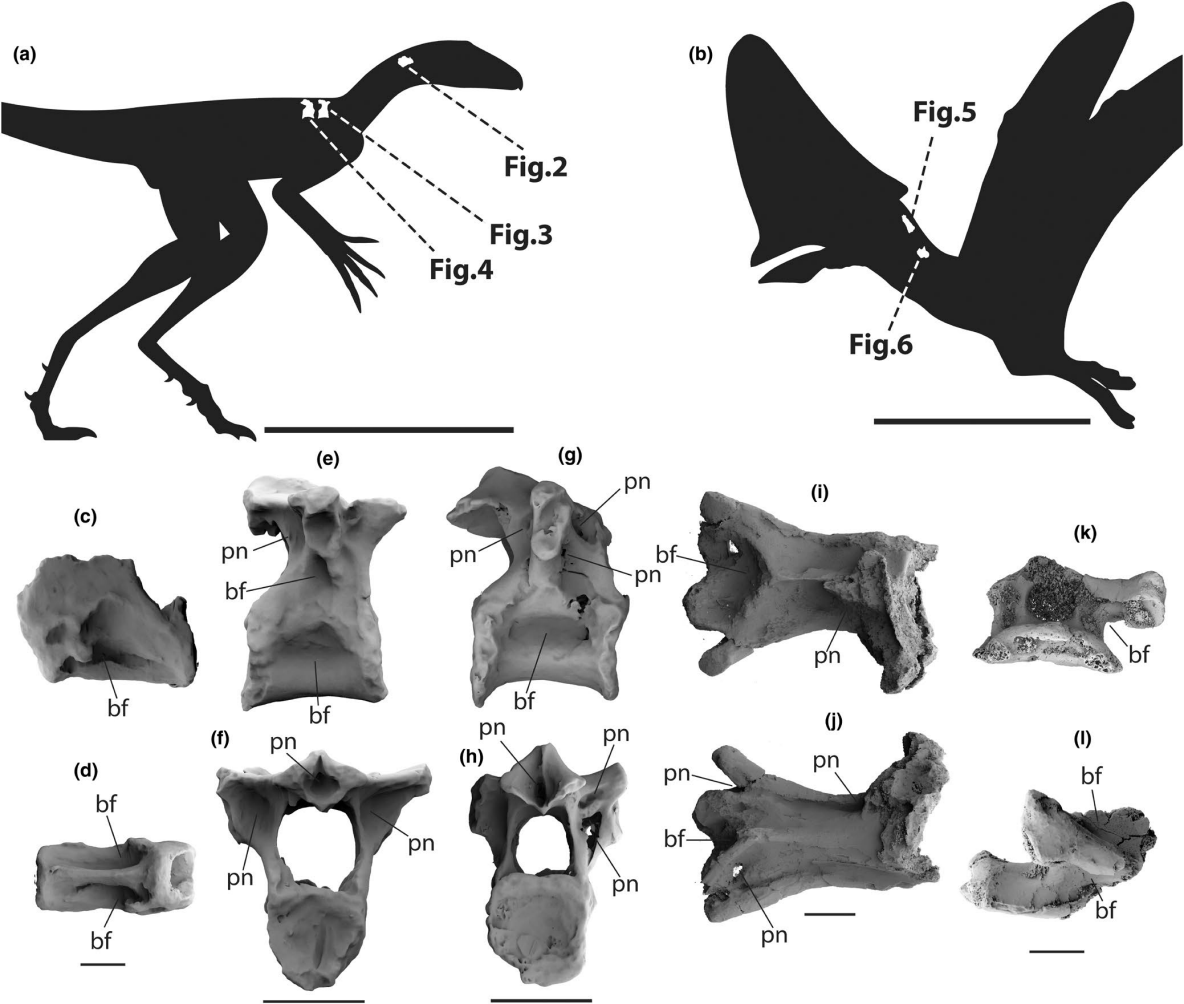
Vertebrae of the lagerpetid pterosauromorph *Venetoraptor gassenae* (CAPPA/UFSM 0356; a, c–h) and the tapejarine pterosaur *Caiuajara* sp. (LPP‐UFRN 3001; b, i–l) showing surficial pneumatic features (a–l). Silhouettes indicate vertebral positions and corresponding figures (a, b). (c, d) Cervical vertebra of *Venetoraptor* in left lateral (c) and ventral (d) views. (e, f) First dorsal vertebra in right lateral (e) and posterior (f) views. (g, h) Second dorsal vertebra in right lateral (g) and posterior (h) views. (i, j) Anterior cervical vertebrae of *Caiuajara* in dorsal (i) and ventral (j) views. (k, l) Cervical vertebra in cranial (k) and left lateral (l) views. bf, blind fossa; pn, external pneumatic foramen. All vertebrae are 3D reconstructions from micro‐CT scans. Scale bars: a–g = 10 mm; h–j = 5 mm; k–l = 500 mm. Silhouettes from Rodrigo T. Müller and Hugo Salais‐López modified from Felipe A. Elias.

CAPPA/UFSM 0356, holotype of *Venetoraptor gassenae*, is a partial skeleton of a single individual, partially articulated (Figure 1a). Comparisons with other lagerpetids (Nesbitt et al., 2009) and dinosauromorphs (Piechowski et al., 2014) suggest the presence of the trochanteric shelf on the femur indicates the specimen is not skeletally immature. However, it is not currently possible to confirm whether the specimen has reached its full size. Our analysis used two well‐preserved anterior dorsal vertebrae (denoted D1 and D2, i.e., first and second dorsals from the front) and one anterior cervical vertebra of CAPPA/UFSM 0356, all described in the original study (Müller et al., 2023).

We compared the data from CAPPA/UFSM 0356 with some pterosaur scans found in the literature, and also scanned a tapejarine specimen for comparison in this study. This tapearine is LPP‐UFRN 3001 (provisional inventory number), an incomplete specimen of *Caiuajara* sp., comprised of two partially preserved cervical vertebrae (one anterior and one middle; Figure 1b). The anterior cervical vertebra lacks part of the neural spine and transverse processes. The middle only preserved the anterior dorsal portion of the centrum and the ventral portion of the neural arch, including the left prezygapophysis. The fusion of the neurocentral suture suggests an advanced ontogenetic stage (Brochu, 1996).

This specimen was loaned by Felipe Pinheiro, Federal University of Pampa (Unipampa) and was temporarily registered at the DINOlab‐UFRN. In the future, the specimen may be permanently registered in a collection closer to where the fossils were found in the state of Paraná, or in some other institution according to the requirements of the curator. We used both vertebrae in our analysis.

[Correction added on 18 September 2025 after first online publication: This section has been revised.]

### 2.3 Locality and horizon

CAPPA/UFSM 0356 was excavated at the Buriol site (29° 39′ 34.2″ S, 53° 25′ 47.4″ W), São João do Polêsine, Rio Grande do Sul state, South Brazil. This outcrop is part of the lower portion of the Candelária Sequence (Horn et al., 2014), within the Santa Maria Supersequence (Zerfass et al., 2003), Paraná Basin. These layers are part of the *Hyperodapedon* Acme Zone of the *Hyperodapedon* Assemblage Zone (Langer et al., 2007), with a maximum deposition age of 233.23 ± 0.73 million years—Carnian, Late Triassic (Langer et al., 2018).

LPP‐UFRN 3001 was collected near Cruzeiro do Oeste municipality, western Paraná state, South Brazil (Basilici et al., 2012), from an outcrop of the Goio‐Erê Formation, Caiuá Group, Bauru Basin (Fernandes & Coimbra, 2000). The age of the Goio‐Erê Formation remains debated, with proposed ages ranging from Turonian–Campanian (Fernandes & Coimbra, 2017) to Coniacian (Milani et al., 2007), or even Aptian–Albian.

[Correction added on 18 September 2025 after first online publication: This section has been revised.]

### 2.4 Anatomical nomenclature

We followed the anatomical nomenclature of previous works (Wilson, 2012; Wilson et al., 2011) for vertebral laminae and fossae (Wilson, 2012; Wilson et al., 2011), and Aureliano et al. (2022, 2023), O'Connor (2004, 2006), Wedel et al. (2000), and Wedel (2003) for pneumatic and apneumatic structures.

### 2.5 Microcomputed tomography (μCT scan)

**FIGURE 2:**
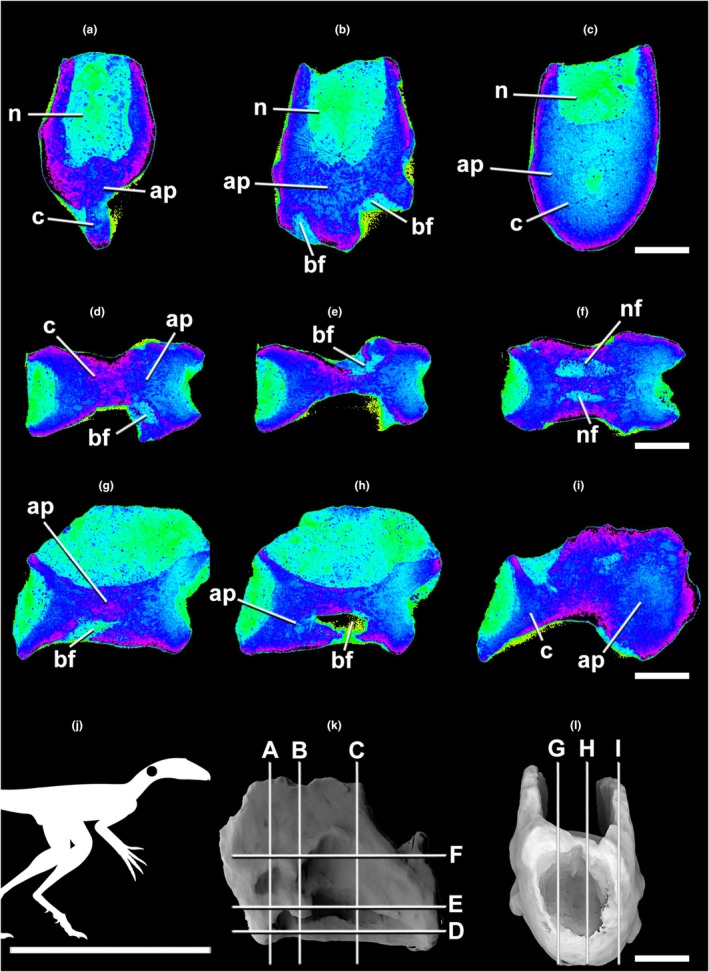
Micro‐CT scan of the lagerpetid pterosauromorph *Venetoraptor* (CAPPA/UFSM 0356) middle cervical vertebra in transverse (a–c), frontal (d–f), and parasagittal (g–i) selected sections. (j) The silhouette shows the position of the axial element. The scans reveal the apneumatized internal architecture of the centrum. Lighter blue indicates lower densities (e.g., sediment and pneumatic cavities). Darker blue and pink illustrate denser structures (e.g., bone tissue). Index of slices in lateral (k) and cranial (l) views. Ap, apneumatic bone; bf, blind fossa; c, centrum; n, neural canal; nf, neurovascular foramen. Scale bar in a–i,k,l = 4 mm; in j = 1000 mm.

**FIGURE 3:**
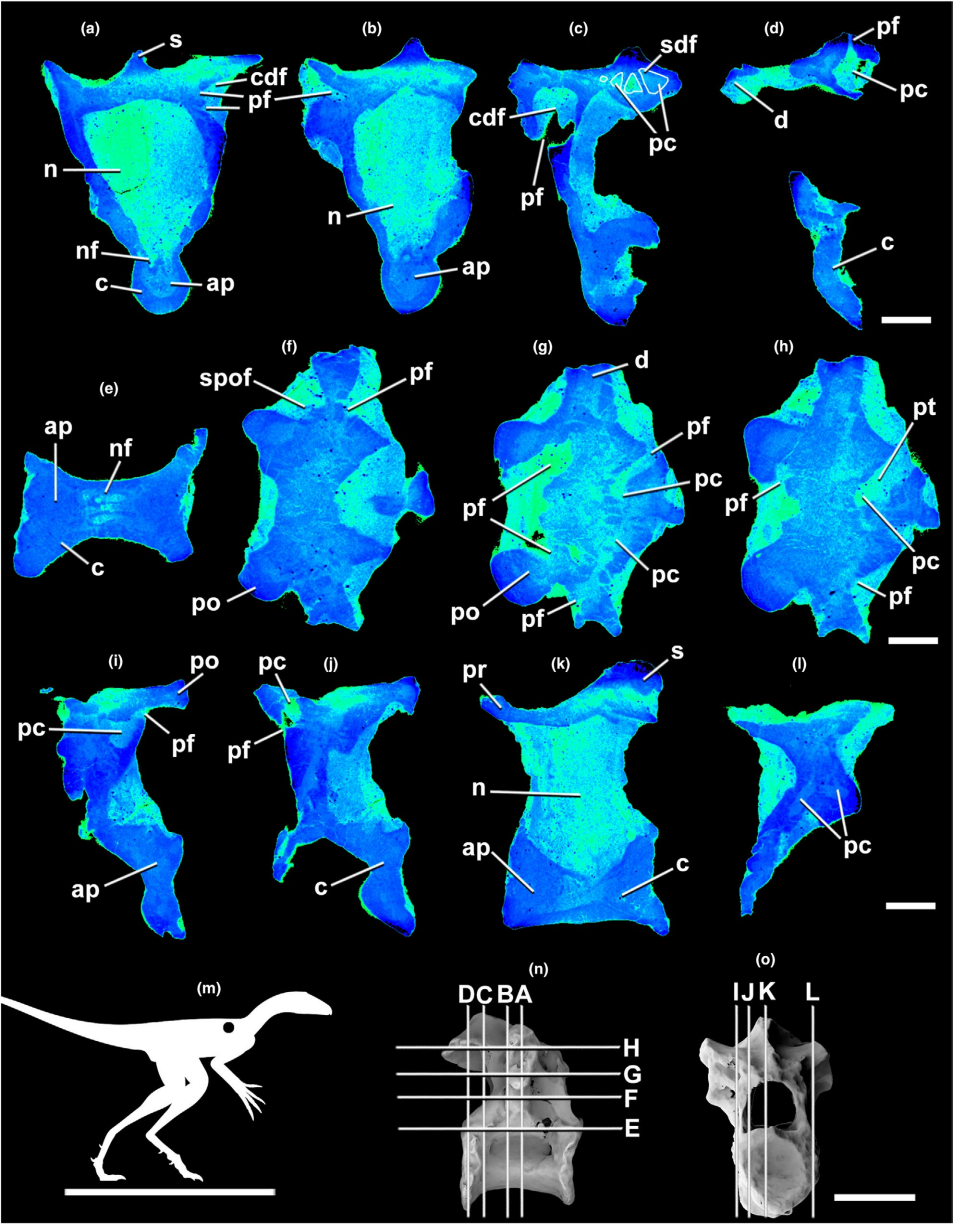
Micro‐CT scan of the lagerpetid pterosauromorph *Venetoraptor* (CAPPA/UFSM 0356) anterior dorsal (1st) vertebra (D1) in transverse (a–c), frontal (d–f), and parasagittal (g–i) selected sections. (j) the silhouette shows the position of the axial element. The scans reveal the pneumatized internal architecture of the neural arch. Lighter blue indicates lower densities (e.g., sediment and pneumatic cavities). Darker blue illustrates denser structures (e.g., bone tissue). The outline in white shows pneumatic chambers. Index of slices in lateral (N) and cranial (O) views. Ap, apneumatic bone; bf, blind fossa; c, centrum; cdf, centrodiapophyseal fossa; d, diapophysis; n, neural canal; nf, neurovascular foramen; pc, pneumatic chambers; pf, pneumatic foramen; po, postzygapophysis; pocdf, postzygacentrodiaphophyseal fossae; pr, prezygapophysis; pt, pneumatic tube; s, neural spine; sdf, spinodiapophyseal fossa. Scale bar in a–i = 5 mm; in j = 1000 mm; in k, l = 10 mm.

**FIGURE 4:**
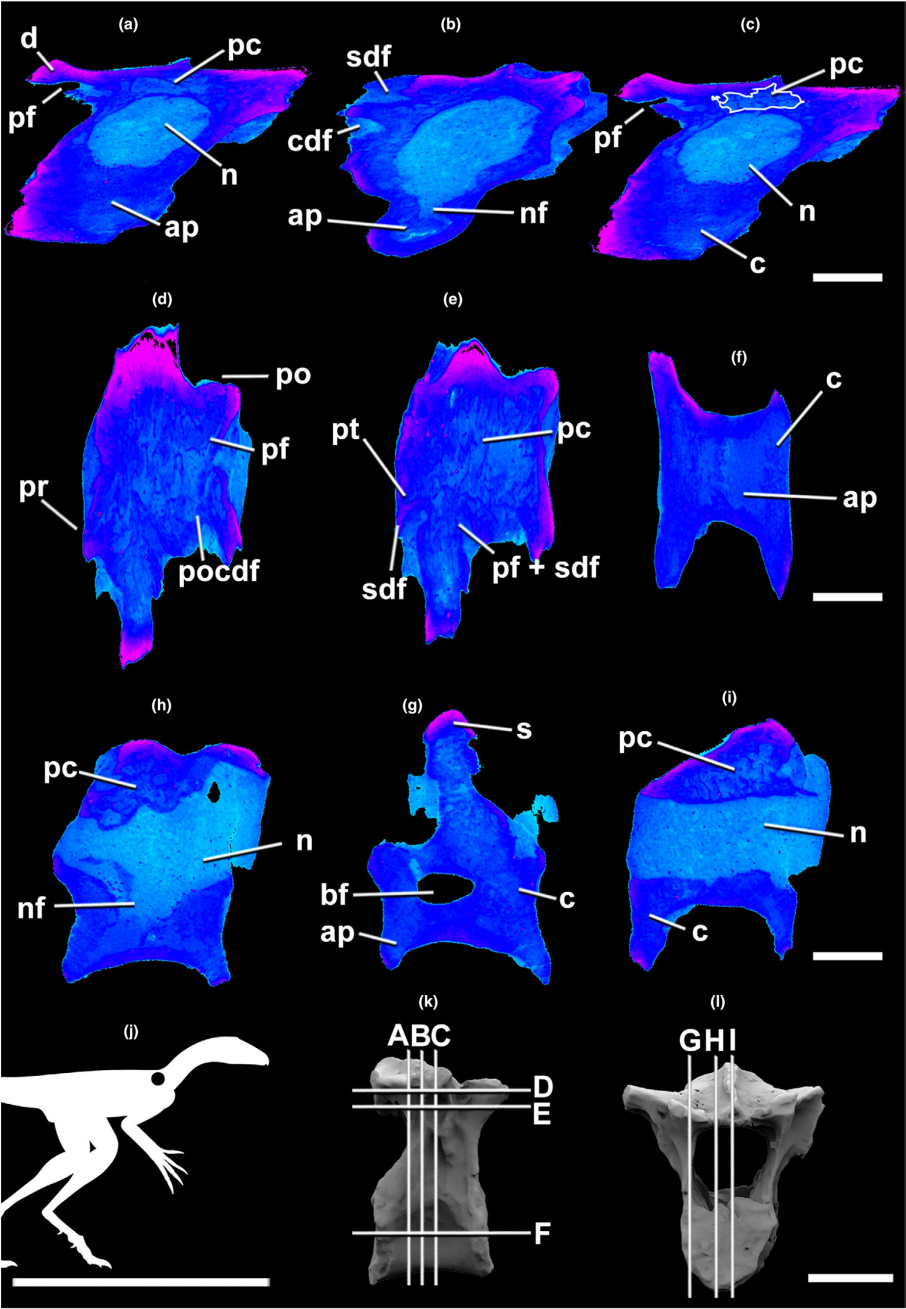
Micro‐CT scan of the lagerpetid pterosauromorph *Venetoraptor* (CAPPA/UFSM 0356) anterior dorsal (2nd) vertebra (D2) in transverse (a–d), frontal (e–h), and parasagittal (i–l) selected sections. (m) The silhouette shows the position of the axial element. The scans reveal the pneumatized internal architecture of the neural arch. Lighter blue indicates lower densities (e.g., sediment and pneumatic cavities). Darker blue and pink illustrate denser structures (e.g., bone tissue). The outline in white shows a pneumatic chamber. Indeces of slices in lateral (k) and cranial (l) views. Ap, apneumatic bone; c, centrum; cdf, centrodiapophyseal fossa; d, diapophysis; n, neural canal; nf, neurovascular foramen; pc, pneumatic chambers; pf, pneumatic foramen; po, postzygapophysis; pr, prezygapophysis; s, neural spine; pt, pneumatic tube; sdf, spinodiapophyseal fossa; spof, spinopostzygapophyseal fossa. Scale bar in a–l = 3 mm; in m = 1000 mm; in n, o = 10 mm.

**FIGURE 5:**
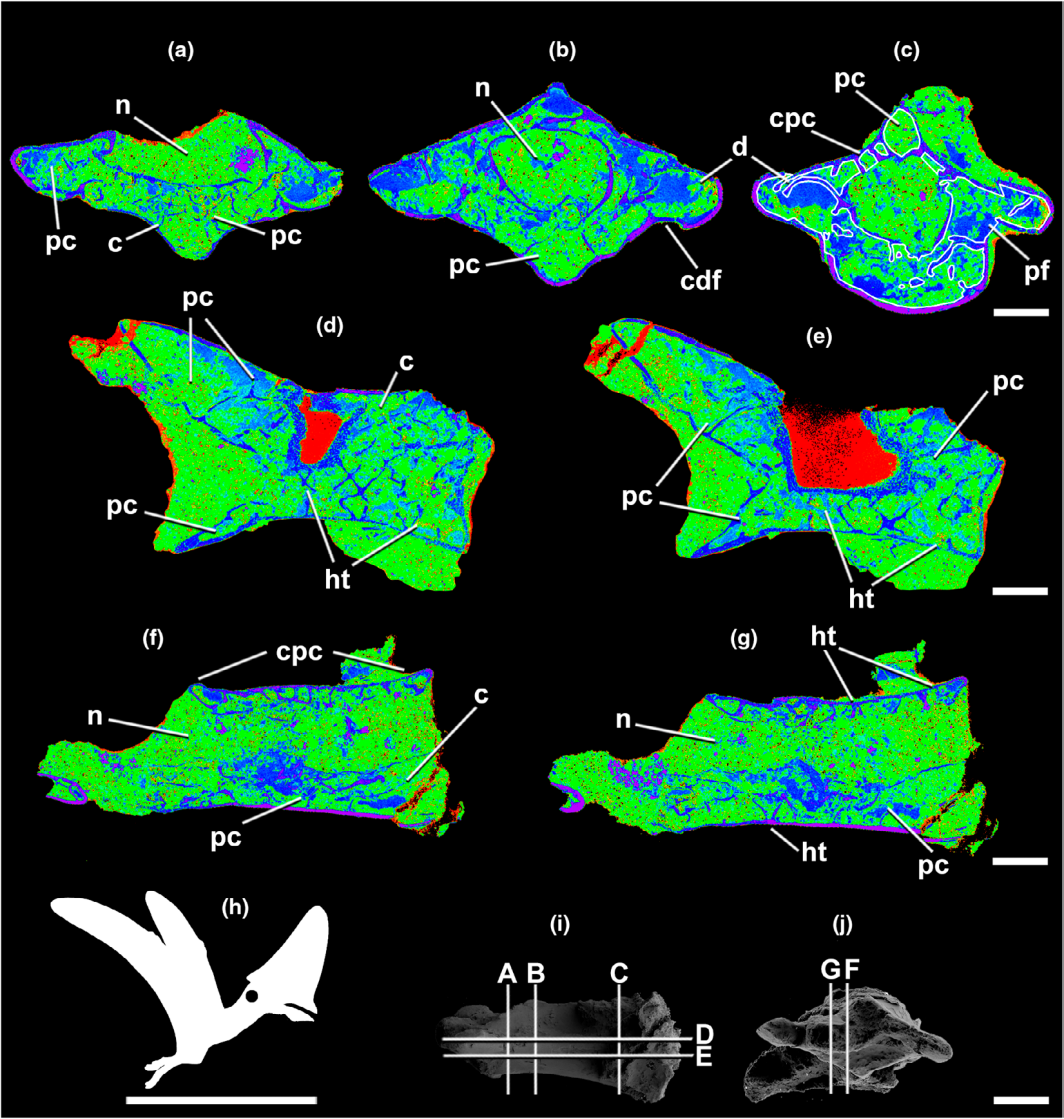
Micro‐CT scan of the tapejarine pterosaur *Caiuajara* (LPP‐UFRN‐3001.a) anterior cervical vertebra in transverse (a–c), frontal (d, e), and parasagittal (f, g) selected sections. H, the silhouette shows the position of the axial element. The scans reveal the intensely pneumatized internal vertebral architecture. Lighter blue and green indicate lower densities (e.g., sediment and pneumatic cavities). Darker blue and purple illustrate denser structures (e.g., bone tissue). Red indicates empty spaces. The outline in white shows pneumatic chambers. The outline in white shows pneumatic chambers. Index of slices in lateral (i) and cranial (j) views. Silhouette from Hugo Salais‐López modified from Felipe A. Elias. C, centrum; cdf, centrodiapophyseal fossa; cpc, cell‐like pneumatic chambers; d, diapophysis; ht, helicoidal trabeculae; n, neural canal; pc, pneumatic chambers; pf, pneumatic foramen. Scale bar in a–g = 3 mm; in h = 1000 mm; in i, j = 5 mm.

**FIGURE 6:**
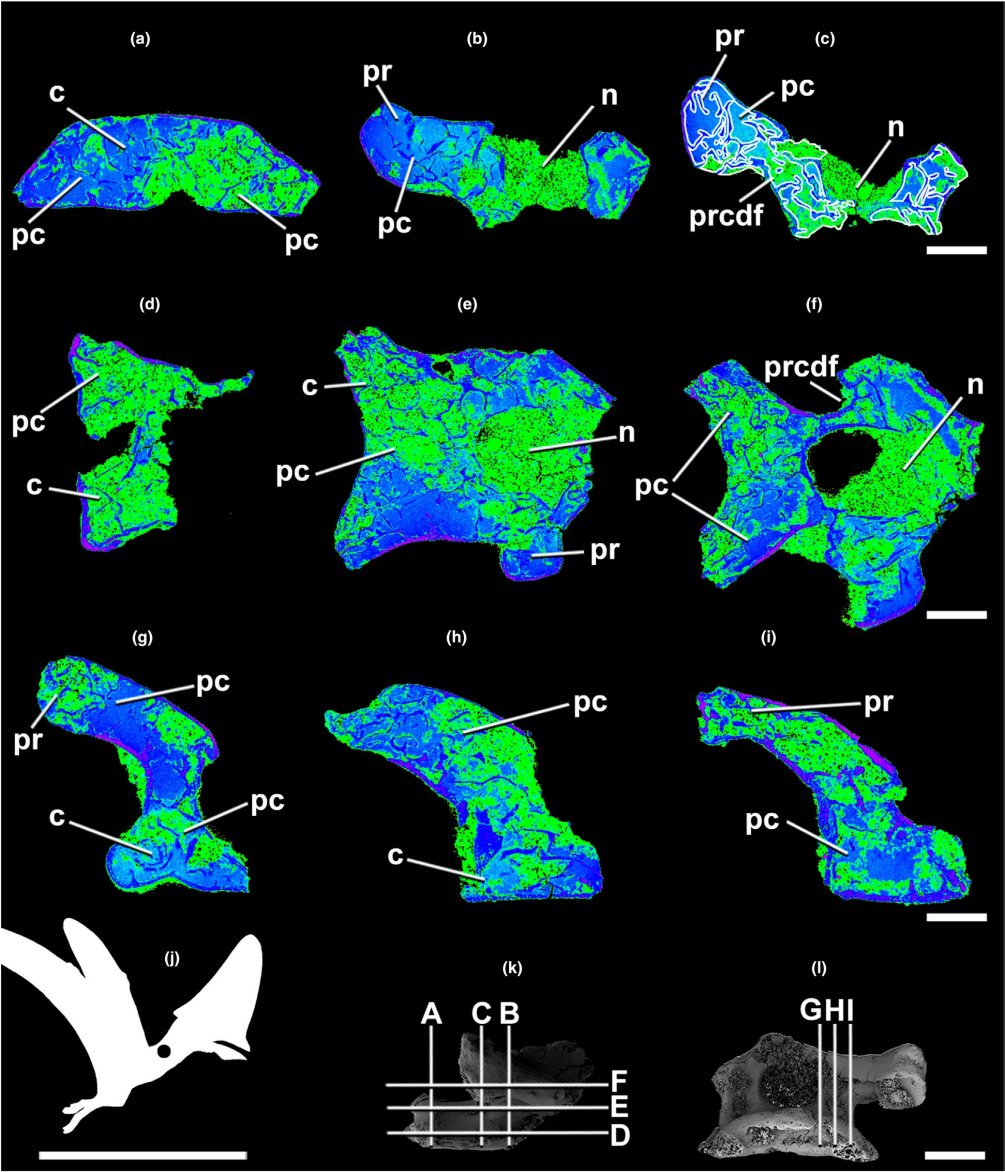
Micro‐CT scan of the tapejarine pterosaur *Caiuajara* (LPP‐UFRN‐3001.b) anterior cervical vertebra in transverse (a–c), frontal (d, f), and parasagittal (g–i) selected sections. (j) The silhouette shows the position of the axial element. The scans reveal the intensely pneumatized internal vertebral architecture. Lighter blue and green indicate lower densities (e.g., sediment and pneumatic cavities). Darker blue and purple illustrate denser structures (e.g., bone tissue). The outline in white shows pneumatic chambers. Index of slices in lateral (k) and cranial (l) views. Silhouette from Hugo Salais‐López modified from Felipe A. Elias. C, centrum; n, neural canal; pc, pneumatic chambers; pr, prezygapophysis; prcdf, prezygacentrodiapophyseal fossa. Scale bar in a–g = 3 mm; in j = 1000 mm; in k, l = 5 mm.

CAPPA/UFSM 0356 was scanned on a Bruker‐Skyscan 1173 microtomographer at the Instituto do Petróleo e dos Recursos Naturais at Universidade Católica do Rio Grande do Sul (PUC‐RS), Porto Alegre, Rio Grande do Sul state, Brazil, using a 130‐kV μ‐focus X‐ray source with a voxel size of 0.15 mm (Figures 1a, 2, 3, 4). LPP‐UFRN 3001 was scanned on a Nikon Metrology XT H 224 ST microtomographer at the Department of Nuclear Energy at the Federal University of Pernambuco (UFPE), Recife, Pernambuco state, Brazil, using a 300‐kV μ‐focus X‐ray source with a voxel size of 0.10 mm (Figures 1b, 5 and 6). We followed the methods described by Aureliano et al. (2020, 2021), Gilbert et al. (2016), Snively and Theodor (2011), and Snively and Miyashita (2012) to elaborate densitometry‐based rainbow color ranges based on the non‐dimensional Hounsfield counts. Our μCT densitometry analysis only highlights relative density differences within each scan and cannot provide absolute bone mineral measurements. The displayed color gradients correspond to variations in X‐ray attenuation, allowing us to separate compact cortical tissue from trabecular regions and pore‐filling matrix; yet, these scales cannot be directly compared among specimens because fossilization and matrix chemistry differ. Consequently, we relied on a qualitative assessment, as mineral infillings and specimen‐specific diagenetic changes make quantitative calculations unreliable. The scans were analyzed with 3D Slicer version 5.2.1 (Fedorov et al., 2012). CT scans are available at Morphosource (https://www.morphosource.org/projects/000520041 and https://www.morphosource.org/projects/000681269).

## 3 RESULTS

Micro‐CT densitometry analysis allowed the observation of fine internal osteological structures of *Venetoraptor* (CAPPA/UFSM 0356) (Figures 2, 3, 4) and *Caiuajara* vertebrae (Figures 5 and 6). Although taphonomic artifacts, such as diagenetic compression and cracks, and low contrast between mineral infills and bone tissue, affected *Venetoraptor* (CAPPA/UFSM 0356) vertebrae, many internal details were still observable. *Caiuajara* (LPP‐UFRN 3001) exhibited better preservation, despite the fragmentary condition, allowing for clearer identification of its internal pneumatic features.

### 3.1 Pneumatic structures in *Venetoraptor* vertebrae

The anterior dorsal and cervical vertebrae of *Venetoraptor* (CAPPA/UFSM 0356) presented a mosaic of pneumatic features. Large, blind lateral fossae (bf) were observed on the analyzed cervical and dorsal vertebrae. The internal architecture of the centra (c) comprises thick cortical walls surrounding a dense apneumatic trabecular matrix, without connections to pneumatic canals, in both cervical and dorsal vertebrae (Figures 2a–i, 3d, e, i–k, 4a–c, f–i). The spinopre‐ and spinopostzygapophyseal fossae (sprf and spof) are not preserved in the cervical but are shallow in D1 and deep in D2, both displaying pneumatic foramina (pf) connected with internal pneumatic chambers (pc) (Figures 3f–h and 4e). Additionally, pneumatic cavities are present ventral to the spinodiapophyseal fossae (sdf) in D1 and D2 (Figures 3c,d,g,h and 4a,b,d,e) and the centrodiapophyseal fossae (cdf) always bear pneumatic foramina (pf) in D1 and D2 (Figures 3 and 4a–d).

### 3.2 Pneumatic structures in *Caiuajara* vertebrae

In *Caiuajara* (LPP‐UFRN 3001), the anterior and middle cervical vertebrae exhibit shallow lateral and centrodiapophyseal fossae (cdf; Figures 5 and 6). The lateral fossae on the anterior cervical are blind and anteroposteriorly elongated (Figure 5a,b), while the centrodiapophyseal fossae (cdf) have pneumatic foramina (pf) connected with internal pneumatic chambers (pc) (Figure 5c). Internal pneumatic chambers (pc) fill the entire vertebral body in both vertebrae (Figures 5a–g and 6a–i). The internal architecture of the anterior cervical comprises a helically arranged trabecular matrix across the entire vertebral body (Figure 5d–g). There are also cell‐like pneumatic chambers (cpc) dorsal to the neural arch in the anterior cervical vertebra (Figure 5c,f).

## 4 DISCUSSION

**FIGURE 7:**
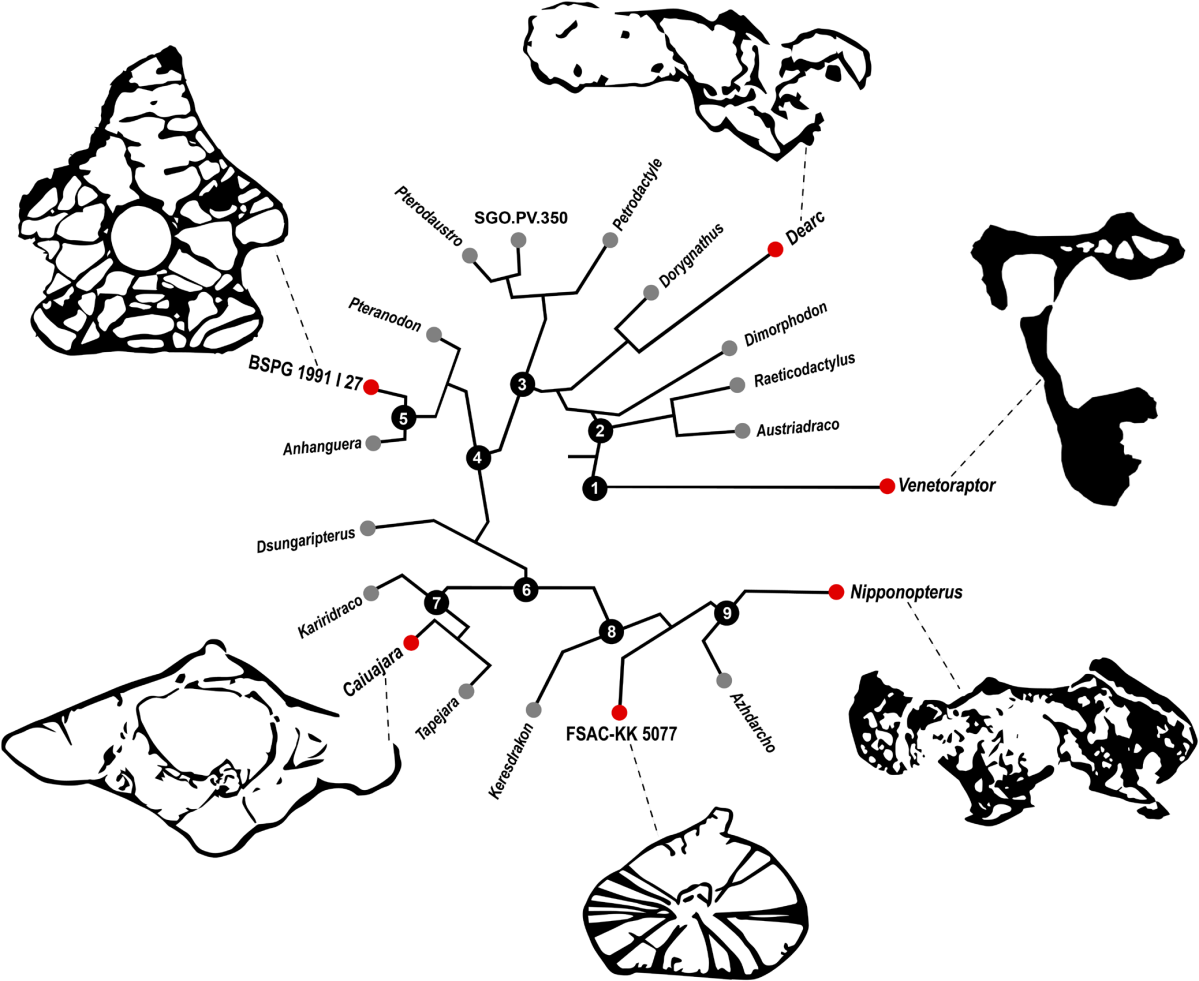
Pneumatized pterosauromorph vertebrae and their phylogenetic context. Taxa with unambiguous PSP described through external features (e.g., large foramina, internal chambers exposed from breaks) are represented by gray circles. Taxa with PSP described through CT scans are indicated by red circles (high‐contrast images). Clades represented are: 1, Pterosauromorpha; 2, Pterosauria; 3, Pterodactyloidea; 4, Ornithocheiroidea; 5, Anhangueridae; 6, Azhdarchoidea; 7, Tapejaromorpha; 8, Azhdarchomorpha; 9, Azhdarchidae. Specimens listed: *Anhanguera* (Kellner & Tomida, 2000), *Austriadraco* (Dalla‐Vechia, 2021), *Azhdharco* (Averianov, 2010), BSPG 1991 I 27 (Buchmann et al., 2021), *Caiuajara* (this study), *Dearc* (Jagielska et al., 2022), *Dimorphodon* (Holgado, 2020), *Dorygnathus* (Holgado, 2020), *Dsungaripterus* (Britt, 1993), FSAC‐KK 5077 (Williams et al., 2021), *Kariridraco* (Cerqueira et al., 2021), *Keresdrakon* (Kellner et al., 2019), *Nipponopterus* (Zhou et al., 2025), *Petrodactyle* (Hone et al., 2023), *Pteranodon* (Britt, 1993), *Pterodaustro* (Holgado, 2020), *Raeticodactylus* (Butler et al., 2009), SGO.PV.350 (Alarcón‐Muñoz et al., 2020), *Tapejara* (Eck et al., 2011), *Venetoraptor* (this study). Topology modified after Zhou et al. (Zhou et al., 2025). All in sagittal view. Not to scale.

The microanatomical analysis revealed an increase in pneumatic complexity (in both vertebral centra and neural arches) from *Venetoraptor* to *Caiuajara* (Figure 7). The trabecular architecture in the dorsal vertebrae of *Venetoraptor* presents a mosaic of characteristics similar to other archosaurs, especially early sauropodomorph and herrerasaurid dinosaurs. The thick cortical wall of *Venetoraptor* vertebrae, filled with low‐density, haphazardly oriented trabecular arrays, is a condition shared with *Alligator* (NHMUK RR 1975.1423), *Varanus* (NHMUK RR 1934.9.2.2) (Butler et al., 2012), and the saurischian dinosaurs *Buriolestes* (CAPPA/UFSM 0035), *Pampadromaeus* (ULBRA‐PV016) and *Gnathovorax* (CAPPA/UFSM 0009) (Aureliano et al., 2022). This differs from the highly dense trabecular matrices seen in the rhynchosaur *Stenaulorhynchus* (NHMUK R36618), *Erythrosuchus* (NHMUK R3592), Phytosauria indet. (NHMUK OR38072), Aetosauria indet. (NHMUK OR38070), and *Silesaurus* (ZPAL Ab III) (Butler et al., 2012). The pneumatic structures in the neural arches of *Venetoraptor* are very similar to the ones seen in the unaysaurid sauropodomorph dinosaur *Macrocollum* (CAPPA/UFSM 0001b). The internal structures of the *Caiuajara* vertebrae are similar to the delicate, very low‐density trabecular matrices and thin cortices of the pterodactyloids Anhanguerinae indet. (SNSB/BSPG 1991 I 27) (Buchmann et al., 2021), Azhdarchiformes indet. (FSAC‐KK 5077) (Williams et al., 2021), and *Nipponopterus* (MDM 349) (Zhou et al., 2025). The same internal structure is shared with the anurognathid ‘*Mesadactylus*’ (BYU 9126) (Smith et al., 2004) and the rhamphorhynchid *Dearc* (NMS G.2021.6) (Jagielska et al., 2022).

It remains uncertain whether the pneumatic condition of *Venetoraptor* is unique among lagerpetids or shared with other members of the clade. Axial remains from the pre‐sacral column of lagerpetids are scarce and have only been described for *Lagerpeton* and *Ixalerpeton*. In addition, some specimens of the putative lagerpetid *Scleromochlus* preserve the axial series. However, the poor preservation of these specimens hinders the observation of the presence or absence of laminae and fossae (Foffa et al., 2024). The holotype of *Lagerpeton* preserves a series of mid‐to‐posterior dorsal vertebrae. Although these elements are poorly preserved, making the identification of laminae difficult, deep fossae can be observed on the lateral surfaces of the centra, a condition that is shared with *Venetoraptor* (Müller et al., 2023). Regarding *Ixalerpeton*, the holotype preserves pre‐sacral vertebrae 6–20. Unlike *Venetoraptor*, the neural arches of these vertebrae lack laminae (Cabreira et al., 2016). Considering this variation, some degree of variation in vertebral pneumaticity can be expected within Lagerpetidae.

### 4.1 Remarks on the evolution of invasive air sacs in pterosauromorphs

Evidence of unambiguous PSP is restricted to pterosauromorphs and saurischian dinosaurs (Aureliano et al., 2023, 2024; Benson et al., 2012; Buchmann et al., 2021; Butler et al., 2009, 2012; Smith et al., 2021; Wedel, 2009). The pneumatization of the postcranial skeleton evolved relatively earlier in pterosauromorphs than in theropods and sauropodomorphs. The earliest saurischian dinosaurs did not feature PSP (Aureliano et al., 2022), but *Venetoraptor* demonstrates that invasive air sacs were present in the pterosauromorph lineage before the appearance of the earliest pterosaurs. Also notable is the intensity and complexity of vertebral pneumatization (Figure 7), with diverticula invading the entire vertebral structure as early as in *Raeticodactylus* (Butler et al., 2009) and *Austriadraco* (Dalla‐Vechia, 2021), based on external observations on large openings allowing the visualization of internal camerae. In the rhamphorhynchid *Dearc* (NMS G.2021.6) (Jagielska et al., 2022), a CT scan revealed a thin cortex surrounding the vertebra and the low‐density trabecular architecture sustaining the vertebral body. An analog to this microanatomical adaptation in theropods would only appear in coelurosaurs and noasaurids (Aureliano et al., 2024; Brum et al., 2018; Gianechini & Zurriaguz, 2021; Smith et al., 2021), and in sauropods (late‐branching titanosaurs) (Aureliano et al., 2021; Cerda et al., 2012; Zurriaguz, 2024). Such an early development of PSP would have improved ventilatory efficiency (thus enabling greater oxygen delivery) and diminished body density in pterosauromorphs, facilitating the evolution of flight in early pterosaurs by the Late Triassic. The presence of PSP in *Venetoraptor* provides evidence that the development of this feature in pterosauromorphs occurred before the evolution of active flight. Previously, this phenomenon was only observed in theropods, where non‐avian species exhibited pneumaticity long before the emergence of Paraves (Aureliano et al., 2024; Gianechini & Zurriaguz, 2021; Britt, 1993; O'Connor & Claessens, 2005; Smith et al., 2021; Wedel, 2009). However, since the earliest known pterosaurs, such as *Raeticodactylus*, already possessed well‐developed flying apparatuses (Butler et al., 2009), PSP was thought to be exclusively linked to flight capabilities. This adaptation could also have been crucial for the pterosaurs evolving flight before theropods in the evolutionary history, and maintaining niche dominance throughout the Mesozoic (Benson et al., 2014).

Future investigations scanning vertebrae of more basal pterosaur taxa, particularly early diverging non‐pterodactyloids and transitional forms, would significantly advance our understanding of the evolutionary trajectory of postcranial pneumaticity across Pterosauria (Buchmann & Rodrigues, 2019; Butler et al., 2009; Claessens et al., 2009). While such comprehensive scanning was not feasible for this publication due to specimen availability and preservation constraints, targeted analyses of plesiomorphic pterosaurs could illuminate the timing and sequence of pneumatic character acquisition, potentially revealing whether the complex trabecular architectures observed in derived taxa were present ancestrally or evolved iteratively (Aureliano et al., 2024; Buchmann & Rodrigues, 2019). These future analyses would provide critical interpolation points for mapping pneumatic evolution across the pterosaur phylogenetic tree.

### 4.2 Remarks on the helicoidal trabeculae in azhdarchoid vertebrae

Helically arranged trabeculae were first identified in an azhdarchiform cervical vertebra (FSAC‐KK 5077; Figure 7) from the Late Cretaceous of Morocco (Williams et al., 2021). These radial trabeculae likely enhanced the stability of the neck's structure, providing protection to the neural canal. Here, we report similar structures in the cervical vertebrae of the tapejarine *Caiuajara* sp. (Figure 5) from the Early Cretaceous of Brazil. The identification of a helicoidal trabecular architecture in *Caiuajara* suggests that this trait was present in Azhdarchoidea prior to the emergence of the Azhdarchomorpha, occurring in the Tapejaridae and preceding the evolution of this bone tissue arrangement by several million years. Future analyses across a broader range of taxa will help determine whether this could be a potential plesiomorphy for Azdarchoidea.

## 5 CONCLUSION

The study of the evolution of air sacs in pterosauromorphs provides important insights into the adaptations that facilitated flight in this extinct clade. Our findings indicate that the presence of invasive diverticula branching from air sacs, which enhance respiratory efficiency and reduce body density, predates the evolutionary origin of pterosaurs. Through microcomputed tomography analysis of the lagerpetid pterosauromorph *Venetoraptor gassenae* and the tapejarine pterosaur *Caiuajara* sp., and comparisons with other taxa published in the literature, we identified an increase from simple to complex and fully‐pneumatized structures within the vertebrae. This suggests a relatively faster evolution of postcranial skeletal pneumaticity compared to saurischian dinosaurs. The results demonstrate that *Venetoraptor* exhibited early signs of postcranial skeletal pneumaticity, indicating that such adaptations were already present in the lineage leading to pterosaurs before the evolution of flight in Pterosauromorpha. This evolutionary trajectory likely provided crucial advantages via enhanced ventilation, reduced body density, and increased bone's mechanical strength, which would have been essential for the successful evolution of powered flight.

## AUTHOR CONTRIBUTIONS

T.A. and A.M.G. idealized the study. T.A., A.M.G., and L.K. conducted the microcomputed tomography. R.T.M. collected and prepared the specimen of *Venetoraptor*. T.A. prepared the specimen of *Caiuajara*. T.A. and B.H. analyzed the data. Everyone discussed the results and wrote the manuscript.

## CONFLICT OF INTEREST STATEMENT

The authors declare there are no conflicts of interest.

## Data Availability

The data that support the findings of this study are openly available in Morphosource at https://www.morphosource.org/.
